# Factors associated with different smoking status in European adolescents: results of the SEYLE study

**DOI:** 10.1007/s00787-017-0980-4

**Published:** 2017-04-06

**Authors:** Raphaela Banzer, C. Haring, A. Buchheim, S. Oehler, V. Carli, C. Wasserman, M. Kaess, A. Apter, J. Balazs, J. Bobes, R. Brunner, P. Corcoran, D. Cosman, C. W. Hoven, J. P. Kahn, H. S. Keeley, V. Postuvan, T. Podlogar, M. Sisask, A. Värnik, M. Sarchiapone, D. Wasserman

**Affiliations:** 1 Addiction Help Services BIN, Innsbruck, Tyrol Austria; 2 Institute of Psychology, University of Innsbruck, Innsbruck, Austria; 3 Psychiatry and Psychotherapy B, State Hospital Hall in Tirol, Tyrol, Austria; 4 National Centre for Suicide Research and Prevention of Mental Ill-Health (NASP), Karolinska Institute (KI), Stockholm, Sweden; 5 WHO Collaborating Centre for Research, Methods Development and Training in Suicide Prevention, Stockholm, Sweden; 6 Department of Child and Adolescent Psychiatry, New York State Psychiatric Institute, Columbia University, New York, USA; 7 Department of Health Sciences, University of Molise, Campobasso, Italy; 8 Section for Disorders of Personality Development, Centre for Psychosocial Medicine, University of Heidelberg, Heidelberg, Germany; 9 Orygen Youth Health, Melbourne, Australia; 10 Feinberg Child Study Centre, Schneider Children’s Medical Centre, Tel Aviv University, Tel Aviv, Israel; 11 Vadaskert Child and Adolescent Psychiatric Hospital, Budapest, Hungary; 12 Institute of Psychology, Eötvös Loránd University, Budapest, Hungary; 13 Department of Psychiatry, School of Medicine, Centro de Investigación Biomédica en Red de Salud Mental, CIBERSAM, University of Oviedo, Oviedo, Spain; 14 National Suicide Research Foundation, Cork, Ireland; 15 Clinical Psychology Department, Iuliu Hatieganu University of Medicine and Pharmacy, Cluj-Napoca, Romania; 16 Department of Epidemiology, Mailman School of Public Health, Columbia University, New York, USA; 17 Department of Psychiatry and Clinical Psychology, CHRU de NANCY, Université H. Poincaré, Nancy, France; 18 Pole 6, Centre Psychthérapique de Nancy, Université de Lorraine, Nancy, France; 19 Slovene Centre for Suicide Research, Andrej Marušicˇ Institute, University of Primorska, Muzejski trg 2, 6000 Koper, Slovenia; 20 Estonian-Swedish Mental Health and Suicidology Institute, Tallinn, Estonia; 21 Institute of Social Work, Tallinn University, Tallinn, Estonia

**Keywords:** SEYLE, Adolescent smoking, Mental health, Family problems, Behavioral problems, Substance use

## Abstract

Early onset and long-term smoking are associated with physical and psychological health problems. The aim of the presented analysis was to investigate risk and influencing factors for different smoking status in a big sample of European adolescents. In the context of the “saving and empowering young lives in Europe” (SEYLE) study we surveyed 12,328 adolescents at the age of 13–17 from 11 countries. The survey took place in a school-based context using a questionnaire. Overall 58% reported the onset of ever-smoking under the age of 14 and 30.9% smoke on a daily basis. Multinomial logistic regression model showed significant positive associations between adolescent smoking and internalizing problems (suicidal behavior, direct self-injurious behavior, anxiety), externalizing problems (conduct problems, hyperactivity, substance consumption) and family problems (parental substance consumption, broken home). Our data show that smoking among adolescents is still a major public health problem and adolescents who smoke are at higher risk for mental problems. Further, adolescent smoking is associated with broken home families and parental behaviors. Therefore, early preventive measures are necessary not only for adolescents, but also for their parents.

## Introduction

Tobacco smoking among adolescents is a major public health problem and a global concern [1]. Early onset and long-term smoking are associated with physical and psychological health problems [2] and tobacco is still known as one of the most frequently consumed substances in adolescents [3]. Further, many studies reported that adolescent tobacco use is predictive of psychiatric and psychological health problems like depression, substance consumption, as well as personality disorders [4–7]. Thus, it is necessary to clarify possible risk factors and coherences.

It is universally accepted that the familiar environment and especially parental behavior has an influence on child development and adolescent achievement. Some studies already investigated the relationship of family substance use and the consequences on adolescent legal and drug consumption [8–14]. Bauman and colleagues [15] already claimed not to underestimate the influence of parental smoking on adolescent smoking behavior and urge a new explanation for the association between parental and adolescent smoking behavior. Although findings across various studies showed only weak and inconsistent associations between parental and adolescent smoking, which is most probably due to methodological issues [16], stronger connections between parental smoking and adolescent smoking have been described recently [17–19]. Some studies found that family structure (like intact vs. stepfamilies) is significantly associated with smoking and could be seen as a serious risk factor [20, 21].

Further, several studies ascertained strong significant associations between adolescent smoking and substance consumption like alcohol or drug use [22–24].

Some previous studies reported a strong association between adolescent substance use and attention-deficit/hyperactivity disorder (ADHD) or conduct problems [25–28]. Adolescents suffering from hyperactivity symptoms and ADHD are at increased risk of substance use problems, especially alcohol and nicotine consumption [29, 30].

The association of smoking behavior and suicidality, as well as anxiety symptoms has already been demonstrated in several studies [31–41].

With the implementation of non-suicidal self-injury disorder in the fifth version of the Statistical and Diagnostic Manual of Mental Disorders (DSM-5) the issue around self-injurious behaviors receives an actual impact. In adolescents self-injurious behaviors (SIB) constitute a serious health problem and a major risk factor for future suicidal ideation, suicide attempts and suicide [42–44].

By now different opinions exist regarding the association of SIB and substance abuse among adolescents. Some studies report significant correlations between both [45–48], arguing that similar psychological processes underlie these behaviors [49] or that substance use helps individuals to habituate to SIB [50].

Similarly opposite opinions exist regarding the relationship of adolescent smoking and SIB. Some investigations also established a correlation of smoking and SIB in adolescents [51–53]. Brunner and colleagues [54] recently reported an obvious association between adolescent smoking and direct self-injurious behavior (D-SIB). Others, however, ascertained no significant association between smoking and SIB in both genders [52] or at least in boys [55, 56].

Those earlier findings were either investigated with smaller sample sizes, adults or children, or they were inconsistent along different studies. Based on this fact and various claims on preventive measures regarding juvenile smoking it is necessary to clarify actual influencing and mediating factors such as family problems (broken home families, parental smoking and substance use), internalizing problems (emotional symptoms, anxiety, previous suicide attempts) and externalizing problems (conduct problems, hyperactivity, alcohol and drug consumption) on the basis of an adequate, representive sample of youths.

The present analysis investigates risk and influencing factors for different smoking status in European adolescents. We hypothesized that the risk for adolescent smoking will be considerably increased in adolescents with internalizing problems (anxiety, emotional symptoms, previous suicide attempts, D-SIB), externalizing problems (conduct problems, hyperactivity, substance use like alcohol or drug consumption) and family problems (parental smoking, family drunkenness, broken homes). We also assumed that the sooner adolescents start smoking and with increasing frequency, the more serious problems emerge.

## Methods

### Participants

The multi-center study “Saving and Empowering Young Lives in Europe” (SEYLE) was initiated to evaluate school-based prevention measures for risk behaviors and suicidality in youths. Study design and characteristics of the sample have already been published [57, 58]. In total 168 schools, comprising a sample of 12,395 adolescents from 11 countries (i.e., Austria, Estonia, France, Germany, Hungary, Ireland, Israel, Italy, Romania, Slovenia and Spain), with Sweden as the coordination center, were included. In each country eligible schools were randomly selected and ethical permissions were obtained from local ethical committees. Sixty-seven of the 12,395 participating adolescents were excluded, based on missing relevant data and thus a total of 12,328 adolescents [mean age: 14.9 years, range 13–17; number of female/male participants: 6799 (55.2%)/5529 (44.8%)] were included in further analyses.

### Questionnaire

Baseline evaluations were conducted between October 2009 and December 2010. The baseline structured self-report questionnaire was submitted to adolescents aged between 13 and 17. It contained socio-demographic items (e.g., sex, age, religious affiliation, etc.), risk behaviors and mental symptoms. Family setting was assessed by the question: “We would like to know about your family. Please answer this question about your home, where you live permanently or most of the time and put down the people who live with you at your home”. Questions from the Global school-based student health survey (GSHS; [59]) were applied to determine risk behaviors, such as alcohol or drug consumption. The following item was used to assess alcohol consumption in youths: “How often do you have a drink containing alcohol? For example, 0.33 l beer or cider; glass of wine or 4 cl of strong alcohol” and drug use was investigated by the question: “During your life, how many times have you ever used drugs”. Alcohol consumption of family members was assessed by: “Have you ever seen a family member when they are drunk?”. GSHS-Items were also used to identify adolescent and parental smoking behavior. Juvenile smoking behavior was assessed by the questions “Have you ever smoked cigarettes?” and “How many cigarettes did you smoke per day, during the last 6 months?”, whereas parental smoking behavior was gathered by the question “Which of your parents or guardians use any form of tobacco?”. Emotional symptoms, conduct problems and hyperactivity were investigated by questions of the Strengths and Difficulties Questionnaire (SDQ, [60]). Anxiety was assessed using the Zung Self-Rating Anxiety Scale (SAS; [61]) and suicidality (i.e., previous suicide attempts) was ascertained employing the Paykel Suicide Scale (PSS; [62]).

There are various terms to define self-injurious behaviors such as non-suicidal self-injury (NSSI), deliberate self-harm (DSH) and direct self-injurious behavior (D-SIB). NSSI refers to deliberately inflicted damage to one’s body without suicidal intent [63]. The term deliberate self-harm is a more comprehensive term for self-injurious behaviors [64]. DSH is defined as intentional self-injury or self-poisoning irrespective of with or without suicidal intent [65]. DSH also includes indirect damage to one’s body such as severe substance abuse or taking overdose [54]. The term DSH so far was used mainly within Europe and Australia, whereas many researchers within Canada and the United States prefer the term NSSI [64].

The present paper focuses on D-SIB, which is defined as self-inflicted damage to an individual’s body on purpose, regardless of with or without suicidal intent. It includes direct damage such as self-cutting, -burning, -biting, -hitting, and skin damage by other methods [54]. To investigate adolescents engaging in D-SIB, a 6-item questionnaire was used. This 6-item questionnaire is based on the 9-item Deliberate Self-harm Inventory (DSHI) questionnaire from Bjärehed and Lundh [66], which is a shortened version of the 16-item DSHI by Lundh et al. [67] that originated from the original 17-item DSHI by Gratz [68]. This modified version tends to assess direct self-injurious behavior (D-SIB) to one’s body only and self-injurious acts were combined to shorten and simplify the measure. However, it contains the same facets on frequency, severity and duration [54].

### Statistical analysis

Statistical analysis was performed using IBM SPSS and GraphPad Prism.

Dichotomous variables were constructed for alcohol consumption (at least twice a week vs. no to low use), drug consumption (at least three times in life vs. no to low use), family drinking (seen family member drunk at least sometimes vs. never seen family drunk), parental smoking (no parent smokes vs. one parent or both parents smoke), family setting (broken homes—one birth parent with or without step-parents vs. both parent households—both birth parents), and previous suicide attempts (lifetime history of suicide attempts vs. no previous suicide attempts).

Group variables were generated for adolescent smoking status (non-smoker—never have smoked cigarettes; non-daily smoker—have ever smoked or do smoke occasionally but do not smoke daily; daily smoker—smoke daily at least one cigarette) and onset of smoking (younger than 10 years; younger than 14 years; 14 years or older).

The relationship of different adolescent smoking status with internalizing, externalizing and family problems was first analyzed using Chi-square test. Group differences were tested by Kruskal–Wallis *H* test (i.e., nonparametric analysis of variance) followed by post hoc Mann–Whitney *U* tests for group comparisons. Multinomial logistic regression analysis was used to verify the association between different adolescent smoking status and internalizing, externalizing and family problems controlling for covariate risks. The level of statistical significance was predefined at *p* < 0.05.

## Results

Out of 12,328 participating adolescents, 12.5% reported non-daily smoking and 30.9% smoked daily, with the highest rate in Israel (50.4%) and lowest rate in Ireland (13.4%). 58.0% stated the onset of smoking below 14 years (Table 1). Daily smoking was more common in girls (31.8 vs. 29.8%), whereas early onset (<14) was more common in boys (61.6 vs. 55.3%).

**Table 1:** Prevalences for different adolescent smoking status (non-smoker, non-daily smoker, daily smoker), internalizing problems (anxiety, emotional symptoms, previous suicide attempts, D-SIB), externalizing problems (conduct problems, hyperactivity, substance use like alcohol or drug consumption) and family problems (parental smoking, family drunkenness, broken homes)

	Country of pupil											
	Austria (*N* = 952) (%)	Estonia (*N* = 1037) (%)	France (*N* = 1006) (%)	Germany (*N* = 1444) (%)	Hungary (*N* = 1009) (%)	Ireland (*N* = 1096) (%)	Israel (*N* = 1256) (%)	Italy (*N* = 1192) (%)	Romania (*N* = 1140) (%)	Slovenia (*N* = 1170) (%)	Spain (*N* = 1026) (%)	Total (*N* = 12,328) (%)
Female	63.2	54.0	68.3	52.1	58.9	45.3	18.6	68.0	65.4	70.3	48.3	55.2
Male	36.8	46.0	31.7	47.9	41.1	54.7	81.4	32.0	34.6	29.7	51.7	44.8
Non-smoker	49.5	45.4	57.0	51.0	55.9	82.6	44.9	53.8	68.0	48.0	67.7	56.6
Non-daily smoker	18.2	24.8	13.6	12.9	17.5	4.0	4.8	10.8	10.5	14.7	8.1	12.5
Daily smoker	32.2	29.8	29.3	36.2	26.6	13.4	50.4	35.4	21.5	37.3	24.2	30.9
Age of onset <14	51.5	88.9	47.0	68.3	51.8	77.8	57.6	45.9	52.2	45.6	53.8	58.0
Alcohol consumption	16.2	8.3	3.8	9.2	5.8	1.7	10.6	12.5	4.1	8.6	10.0	8.3
Drug use	3.0	5.2	8.9	4.7	3.9	1.3	3.5	4.1	0.3	10.2	5.8	4.6
Parental smoking (at least one parent)	33.9	43.9	45.2	45.7	32.6	35.0	54.4	39.9	50.1	43.7	56.9	44.0
Family drinking (at least sometimes)	52.8	75.8	48.4	53.1	35.6	58.2	48.6	61.8	55.7	56.6	37.4	53.3
Broken homes	19.5	39.8	19.6	25.9	29.1	15.3	24.0	7.6	13.0	16.4	24.3	21.2
Conduct problems	15.7	22.3	17.6	25.0	14.1	19.4	33.6	30.8	20.9	14.6	12.4	21.1
Hyperactivity	18.1	17.6	22.0	17.4	18.9	20.6	18.2	14.3	11.3	12.8	27.5	17.9
Emotional problems	13.1	10.4	18.5	15.7	10.2	10.7	14.1	11.8	13.9	14.6	13.8	13.4
Anxiety	5.6	4.5	13.4	8.9	4.4	6.7	11.3	4.2	8.4	9.6	6.1	7.6
Suicide attempts	1.5	3.2	7.5	6.6	2.4	3.6	10.7	1.3	2.1	3.3	3.1	4.3
D-SIB (any lifetime)	26.8	33.0	38.6	35.5	17.5	20.8	32.7	21.0	20.7	27.3	29.0	27.7

Kruskal–Wallis *H* test and Chi-square test revealed significant differences between defined adolescent smoking groups and internalizing problems (emotional symptoms, suicidal behavior, direct self-injurious behavior, anxiety), externalizing problems (conduct problems, hyperactivity, substance consumption) and family problems (parental substance consumption, broken home; Table 2).

**Table 2:** Differences in internalizing problems (anxiety, emotional symptoms, previous suicide attempts, D-SIB), externalizing problems (conduct problems, hyperactivity, substance use like alcohol or drug consumption) and family problems (parental smoking, family drunkenness, broken homes) between adolescent smoking statuses

	Non-smoker	Non-daily smoker	Daily smoker	H or *χ* ^2^ (*df* = 2)^a^, *p* value
Age (M, SE)	14.76, 0.01	14.95, 0.02	15.14, 0.02	413.90***
Female gender (%)	54.4	57.8	57.3	10.46**
Suicide attempts (%)	1.7	3.1	8.5	276.19***
DSHI-score (M, SE)	0.37, 0.01	0.74, 0.05	1.28, 0.04	863.70***
Anxiety-score (M, SE)	31.59, 0.09	32.55, 0.19	35.10, 0.13	518.20***
Emotional symptoms-score (M, SE)	2.65, 0.03	2.80, 0.06	3.20, 0.04	142.66***
Conduct problems-score (M, SE)	2.02, 0.02	2.43, 0.04	2.95, 0.03	815.01***
Hyperactivity-score (M, SE)	3.15, 0.03	3.77, 0.05	4.26, 0.04	639.20***
Alcohol consumption (%)	2.3	7.4	18.6	829.11***
Drug use (%)	0.4	3.1	12.7	821.63***
Parental smoking (%)	37.4	45.6	55.2	293.50***
Family member drunk (%)	44.3	61.1	65.6	462.29***
Broken home (%)	16.9	25.2	27.3	163.65***

*** *p* < 0.001; ** *p* < 0.01

^a^Kruskal–Wallis *H* test for continuous variables, Chi-square test for dichotomous variables

Significant associations with smoking could be observed for adolescents with direct self-injurious behavior, anxiety, emotional symptoms, conduct problems and hyperactivity. Further, adolescents with previous suicide attempt smoked nearly three times more often than adolescents without previous suicide attempts and adolescents who reported family problems such as parental smoking or living in broken homes reported daily smoking nearly twice as often than adolescents without such family problems. Further, the biggest amount of juvenile daily smokers was assessed in adolescents with alcohol consumption or drug use. Chi-square test revealed significant differences regarding juvenile daily smoking and no or only small differences in the context of non-daily smoking.

Multinomial logistic regression analysis also revealed significant associations of adolescent daily smoking with internalizing problems (emotional symptoms, suicidal behavior, direct self-injurious behavior, anxiety), externalizing problems (conduct problems, hyperactivity, substance consumption) and family problems (parental substance consumption, broken home; Table 3). Significant associations were obtained for the Zung anxiety-score, emotional symptoms and previous suicide attempts. With each increase on the Zung-scale the odds to belong to the daily smoking group than to the non-smoking group rose about 3%. Further, adolescents with previous suicide attempts compared to adolescents with no former suicide attempts had a 1.7 times (OR = 1.69) higher likelihood for belonging to the daily smoking group as to the non-smoking group. Interesting results were found regarding the SDQ-emotional scale. The likelihood to belong to the non-smokers rather than to daily smokers increased about 10% with each rise on the SDQ-emotional scale.

**Table 3:** Multinomial logistic regression analysis for adolescent smoking status (daily, non-daily) controlled for gender, age and country

Smoking status (ref.: non-smoking)	*B*	Non-daily smoking		*B*	Daily smoking	
		Exp (*B*)	CI 95%		Exp (*B*)	CI 95%
Female gender	−0.10	0.91	0.79–1.04	−0.41***	0.67	0.59–0.75
Age	0.30***	1.35	1.23–1.48	0.29***	1.34	1.24–1.44
DSHI-score	0.15***	1.16	1.11–1.22	0.19***	1.21	1.17–1.26
Anxiety-score	0.01	1.00	0.99–1.02	0.03***	1.03	1.02–1.04
Emotional symptoms	−0.06***	0.94	0.90–0.98	−0.10***	0.91	0.88–0.93
Conduct problems	0.13***	1.14	1.09–1.20	0.20***	1.22	1.18–1.27
Hyperactivity	0.10***	1.11	1.07–1.14	0.14***	1.15	1.12–1.18
Suicide attempts (ref.: no)	0.10	1.11	0.72–1.70	0.53**	1.69	1.25–2.30
Alcohol consumption (ref.: no to low)	0.72***	2.10	1.55–2.73	1.53***	4.61	3.71–5.72
Drugs use (ref.: no to low)	1.64***	5.18	2.98–9.00	2.96***	19.29	11.99–31.01
Parental smoking (ref. no)	0.29***	1.33	1.17–1.52	0.51***	1.66	1.50–1.84
Family member drunkenness (ref.: no)	0.47***	1.61	1.41–1.83	0.59***	1.79	1.61–2.00
Broken home (ref.: no)	0.28***	1.33	1.14–1.55	0.36***	1.43	1.26–1.62

*** *p* < 0.001; ** *p* < 0.01

In addition, multinomial logistic regression revealed significant associations of adolescent daily smoking with conduct problems (OR = 1.22), hyperactivity (OR = 1.15), alcohol consumption (OR = 4.61) and drug use (OR = 19.29). With increasing conduct problems and hyperactivity scores the likelihood for belonging to the daily smoking group rose about 20% and 14%, respectively. Furthermore, adolescents who drink alcohol at least twice a week compared to adolescents who drink no or less alcohol had a 4.6 times higher probability to belong to the daily smoking group than to the non-smoking group. Besides, adolescents who used drugs at least three times in their life compared to adolescents who did not or few times even had a 19.3 times higher likelihood to belong to the daily smokers as to the non-smokers. Family problems were also significantly associated with adolescent daily smoking. Multinomial logistic regression analysis revealed significant associations of parental smoking (OR = 1.66), family member drunkenness (OR = 1.79) and living in broken homes (OR = 1.43).

Multinomial logistic regression analysis also revealed smaller but still significant associations of adolescent non-daily smoking with internalizing problems (emotional symptoms, suicidal behavior, direct self-injurious behavior, anxiety), externalizing problems (conduct problems, hyperactivity, substance consumption) and family problems (parental substance consumption, broken home; Table 3). A negative association was also found regarding the SDQ-emotional scale. No significant associations were found between adolescent non-daily smoking and anxiety, as well as previous suicide attempts.

### Adolescent smoking and direct self-injurious behavior

Another association with adolescent daily and non-daily smoking was found for D-SIB.

Chi-square test revealed a significant difference between defined adolescent smoking groups and D-SIB (*χ* ^2^ = 640.208; *df* = 2; *p* < 0.001). In the non-smoking group, 7.9% reported D-SIB, whereas in the daily smoking group 26.5% of the adolescents showed D-SIB. Further, 56.4% of adolescents with D-SIB and only 26.5% of adolescents without D-SIB reported daily smoking (*p* < 0.001).

Further group comparisons revealed statistically significant differences between different adolescent smoking status and D-SIB (*p* < 0.001). The largest differences emerged between the non-smoking group and the daily smoking group (effect sizes; DSHI, *r* = 0.29). Smaller but still significant differences were found between non-smokers and non-daily smokers (DSHI, *r* = 0.12) and between non-daily smokers and daily smokers (DSHI, *r* = 0.14). Non-daily and daily smokers obtained significantly higher mean scores on the DSHI compared to adolescent non-smokers.

Multinomial logistic regression analysis revealed that with each increase on the DSHI the odds of belonging to the adolescent daily or non-daily smoking group than to the non-smoking group rose about 36.5 or 22.8% (*p* < 0.001) for both genders, all age groups and countries. Even after controlling for influencing factors these findings remained significant (Table 3). With increasing D-SIB scores the likelihood of belonging to the daily or non-daily smoking group rather than to the non-smoking group was now about 19 or 15% and still significant (*p* < 0.001).

### Age of onset

**Fig. 1:**
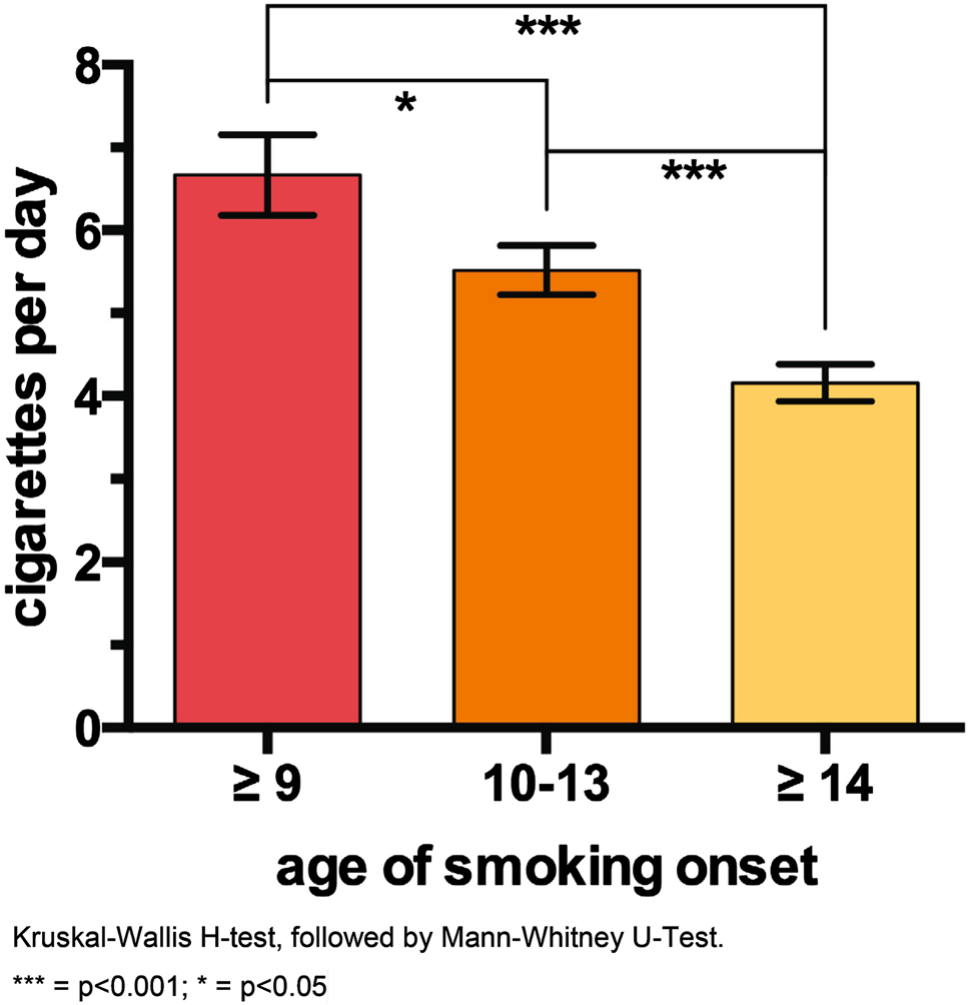
Onset of smoking and cigarettes per day (mean ± standard error)

The sooner adolescents started smoking, the more they smoked at a later point in time and the higher were their mean scores for smoking frequencies. Adolescents who started smoking below 10 years reported 7 cigarettes per day on average, whereas adolescents that reported the onset of smoking under the age of 14 smoked on average 6 cigarettes per day. Adolescents who started smoking only after 14 years reported smoking four cigarettes per day (Fig. 1). Further, the earlier adolescents started smoking the higher the scores they reached on the DSHI score. Group comparisons revealed statistically significant differences between different onset of smoking groups and D-SIB (*p* < 0.001).

## Discussion

We ascertained high prevalence rates for non-daily and daily smoking in a large sample of European adolescents. Also the WHO [3] and OECD [1] indicated high prevalences regarding these topics, therefore corroborating our results. Accordingly, smoking among youths is a serious health problem worldwide.

### Adolescent smoking and internalizing problems

As a very high percentage of youths reported the onset of smoking under 14 years, our data revealed significant differences between different onset of smoking groups and DSHI scores. Many studies state that the early onset of regular tobacco use is predictive of psychiatric and psychological health problems like depression, substance consumption as well as personality disorders [4–7]. Thus, our findings suggest that the early onset of smoking is associated with D-SIB. Our data further verify a coherence of adolescent smoking behavior (especially daily smoking) and D-SIB. Klonsky and Muehlenkamp [49] also argue that self-injurious behavior and substance abuse have to be strongly associated, because both include causing physiological harm to the body. In addition, prior studies found similar correlations between smoking and self-injurious behavior in youths [45–48, 69], arguing that similar psychological processes underlie these behaviors [49] or that substance use helps individuals to habituate to SIB [50].

Further, our data show a relationship between adolescent smoking and psychological issues like anxiety, emotional symptoms and previous suicide attempts. Several studies found similar associations corroborating, for example the relationship between adolescent smoking and anxiety. Patton et al. [39] already stated that subjects reporting high levels of anxiety were twice as likely to be smokers and that anxiety predicted the initiation of experimental smoking [39]. Further, Johnson et al. [36] suggested that early cigarette smoking may increase the risk of certain anxiety disorders during late adolescence. Recently, Cavazos-Rehg et al. [32] reported that smoking cessation is associated with risk reduction of anxiety even among smokers who have had a pre-existing disorder. This is in contrast to the hypothesis that smoking might function as a sort of compensation or self-medication and suggests the assumption of juvenile smoking as a risk factor for anxiety disorders.

Regarding emotional symptoms we found interesting different results. Whereas Chi-square test revealed positive associations between emotional symptoms and adolescents smoking, multinomial logistic regression analysis showed negative associations between these two topics. These findings in turn support the hypothesis that smoking might function as a sort of self-medication. Also Audrain-McGovern and colleagues [70] provide evidence for self-medication processes in the relationship between adolescent smoking and depression.

The association of smoking behavior and suicidality has already been demonstrated in several studies [31, 33–35, 37, 38, 40, 41]. Our findings partially confirm these previous results for a large sample of European adolescents.

To summarize, our data show a relationship between adolescent smoking and psychological issues like anxiety, emotional symptoms, previous suicide attempts and D-SIB.

### Adolescent smoking and externalizing problems

The present paper further suggests that hyperactivity as well as conduct problems increase the risk for smoking in adolescents. Previous studies have documented that adolescents with attention-deficit/hyperactivity disorder (ADHD) or conduct problems are at increased risk of substance use problems [25–28]. Chang et al. [29] found that especially hyperactivity and impulsivity predict early onset of tobacco and alcohol use in youths. Recently, Roberts et al. [30] also stated that specifically hyperactive symptoms were associated with alcohol and nicotine use in young adults, underlining that increased levels of impulsivity are thought to contribute to their increased levels of risk.

Our results further show that there exists a relationship between adolescent smoking and several other risk factors. The strongest associations with juvenile smoking were observed between other substance consumptions like drug and alcohol use. Several studies ascertained strong significant associations between these topics as well and therefore corroborate our results [22–24].

Thatcher and Clarke [71] proposed that psychological dysregulation can be seen as a predictive phenotype for substance use disorders in adolescents. It is characterized by cognitive, behavioral and emotional difficulties in childhood, connecting heritable predispositions and early environmental influences to later substance use disorders. During adolescence, brain circuits including those for motivation, reward and decision-making are not yet fully developed, thereby making this group more susceptible to addiction [72].

### Adolescent smoking and family problems

We further found that family behavior and issues like parental smoking, family drunkenness and living in broken homes influences adolescent smoking behavior. Previous studies confirm the influence of family factors on adolescent smoking behavior. Especially the relationship of parental smoking or tobacco use and adolescent smoking is reported to be very high [17, 73]. Bauman et al. [15] already verified that lifetime parental smoking was as strongly correlated with adolescent smoking as peer smoking. Farkas et al. [74] found that parental smoking cessation keeps adolescents off smoking and that the earlier parents quit smoking, the less likely their children are to become smokers. In addition to that, Almutairi [75] showed that parents had been perceived to be the first source for smoking. Further, Nosa et al. [19] found that exposure to smoking at home seems to be an important risk factor for ever-smoking. Compared to prior findings we found strong effects regarding this topic. Adolescents with smoking parents had a higher likelihood for belonging to the daily smoking group. Furthermore, Griesbach et al. [20] found that family structure (like intact vs. stepfamilies) is significantly associated with smoking among 15-year-olds in seven European countries. Recent findings of Du et al. [21] in multicultural students of Hawaii also show that family structure could be a serious risk factor for smoking among young people. These results demonstrate how important and necessary it is to raise awareness in parents and legal guardians of the damaging influence of their behavior on the smoking behavior of their children to ideally cause lifestyle changes and to make their homes smoke-free.

### Limitations and strengths

It is important to mention that our data do not imply causality, meaning that they do not give an explanation about cause and effect. This limitation of the study is due to the cross-sectional design, where a direct link between determinants and outcome is missing. Concerning this matter further research will be needed. For example, does smoking itself represent a form of self-injurious behavior or does it rather function as a compensation of existing problems?

Another limitation of this analysis would be the missing cross-cultural comparison. Our study comprised adolescents from 11 different countries from all across Europe. Of course there exist cultural differences between the majority of countries, which might also influence adolescent smoking behavior. Future analysis of differences across countries might therefore help to develop country and culture-specific treatment programs. Nevertheless, controlling for country in our regression analysis did not attenuate the effect of the influencing factors we found.

The major strength of this study is its large sample size together with the random selection of schools across 11 representative study sites in Europe. A huge body of literature corroborates our results, most of which are studies from North America. Therefore, to our knowledge, this study is the first investigating factors associated with adolescent smoking in Europe on such a large scale.

## Conclusion

To summarize, the fact is that we have identified very high prevalences and early onset of smoking in European adolescents and that smoking and internalizing problems (emotional symptoms, suicidal behavior, direct self-injurious behavior, anxiety), externalizing problems (conduct problems, hyperactivity, substance consumption) as well as family problems (parental substance consumption, broken home) are consistent and highly associated in an adequate, representative sample of European youths. Further, our data suggest that smoking intensity, which is modulated by both early onset of smoking and family problems, predicts the intensity of externalizing and internalizing problems. Thus, reducing the intensity of smoking from daily to non-daily or preferably no smoking may reduce the intensity of the associated symptoms and might have major clinical and public health consequences.

Therefore, early preventive measures—not only for adolescents but also for their parents—are necessary and essential and should not be neglected. The recently evaluated prevention program “Youth Aware of Mental Health” (YAM) in which a decrease of mental problems, especially suicidal ideations and suicide attempts in high schools was observed [76], could also be developed for young people to prevent early smoking and its problematic outcomes. However, further research will be needed to develop new strategies regarding adolescent smoking and to prevent poor outcomes regarding D-SIB and associated psychological issues in adolescents.
